# Long-Term Outcome of Endothelial Keratoplasty Among Glaucoma Patients and the Risk of Prostaglandin Analogue (Latanoprost) Use on Graft Rejection

**DOI:** 10.3390/jcm14217650

**Published:** 2025-10-28

**Authors:** Majed S. Alkharashi, Mohammed M. Abusayf, Munirah Z. Aldofyan

**Affiliations:** 1 Department of Ophthalmology, College of Medicine, King Saud University, Riyadh 12372, Saudi Arabia; msalkharashi@ksu.edu.sa (M.S.A.); mabusayf@ksu.edu.sa (M.M.A.); 2 King Saud University Medical City, King Saud University, Riyadh 12372, Saudi Arabia; 3 Refractive Surgery and Myopia Research Group, Research Excellence Center in Ophthalmology and Visual Sciences, Department of Ophthalmology, College of Medicine, King Saud University, Riyadh 12372, Saudi Arabia

**Keywords:** DSAEK, endothelial keratoplasty, glaucoma, latanoprost, prostaglandin analog, graft survival

## Abstract

**Background/Objectives**: To evaluate the long-term outcomes of Descemet stripping automated endothelial keratoplasty (DSAEK) in patients with glaucoma and to investigate if the usage of the prostaglandin analog latanoprost increases the risk of graft rejection. **Methods**: This study retrospectively reviewed 65 eyes of 61 patients with glaucoma who underwent DSAEK at King Abdulaziz University Hospital between 2009 and 2024. The risk factors for graft rejection were identified using Kaplan–Meier survival analysis and univariate and multivariate Cox regression models. **Results**: The graft survival rates among patients with glaucoma at 1, 3, and 5 years were 72.4%, 23.1%, and 11.5%, respectively. Latanoprost use was significantly associated with graft failure (71.4% vs. 28.6%, *p* = 0.024). The graft failure was mostly secondary failure (80%, *p* = 0.015) and was often linked to endothelial rejection episodes (62.5%). Other antiglaucoma medications were not associated with graft failure. **Conclusions**: Glaucoma decreases graft longevity after DSAEK. Latanoprost use may further increase the risk of graft rejection by enhancing inflammatory or immune responses. Prospective studies are warranted to confirm these findings.

## 1. Introduction

Endothelial keratoplasty (EK), a widely used effective procedure for managing corneal endothelial failure, is associated with similar visual outcomes and limited complications when compared with penetrating keratoplasty (PKP), especially in patients with no coexisting ocular comorbidities [1,2,3,4,5].

To control intraocular pressure (IOP) in glaucomatous eyes, EK is preferred over PKP [6,7]. Previous studies have demonstrated that glaucomatous eyes are susceptible to graft failure, post-operative complications, and graft dislocation, especially in patients who have undergone glaucoma filtration surgeries showing up to a 50% decrease in graft survival and a two to threefold higher rate of endothelial cell loss compared with non-glaucomatous eyes [8,9,10,11,12,13,14,15,16,17,18].

The risks of glaucoma and glaucoma surgeries have been well documented. However, limited studies have investigated whether antiglaucoma eyedrops, especially prostaglandin analog-based eyedrops, adversely affect graft survival by increasing post-operative inflammation or immune system activation. This study aimed to evaluate EK outcomes among patients with glaucoma, the risk factors for graft rejection, and the effect of topical antiglaucoma drop usage on graft rejection.

## 2. Methods

### 2.1. Study Design and Patients

This retrospective study was conducted at King Abdulaziz University Hospital in Riyadh, Saudi Arabia. The medical records of all patients who underwent Descemet stripping automated EK (DSAEK) between January 2009 and December 2024 were reviewed. All cases with a confirmed diagnosis of glaucoma either pre-existing before DSAEK or newly developed postoperatively and with a minimum postoperative follow-up of three months were included. Eyes with only transient postoperative intraocular pressure (IOP) elevation, without a formal glaucoma diagnosis or the need for medical or surgical glaucoma management, were excluded. Because all available eligible cases during the study period were analyzed, no formal sample-size calculation was performed. This study was approved by the Institutional Review Board of King Saud University Medical City (IRB approval number: E-24-9453; dated 26 January 2025) and performed according to the principles of the Declaration of Helsinki.

### 2.2. Patient Selection and Surgical Approach

All patients with corneal edema who underwent DSAEK and had a history of glaucoma or who developed glaucoma after surgery were eligible for inclusion. The indications included Fuchs’ endothelial dystrophy, pseudophakic bullous keratopathy, aphakic bullous keratopathy, decompensated cornea after silicone oil surgery, decompensated cornea after glaucoma surgery, corneal decompensation after herpetic infection, decompensated penetrating keratoplasty, failed Descemet membrane EK, and cases with no specific known cause. All procedures were performed by board-certified corneal consultants or fellows under supervision. The grafts were inserted using the Busin glide or lens glide technique, depending on the preference of the surgeon. Only the most recent surgery data were included in the analysis for patients who underwent multiple DSAEK procedures on the same eye. This study also included the data of patients who underwent combined procedures (e.g., triple procedures) on the same eyes. Donor tissues were mainly obtained from accredited North American eye banks. After surgery, the patients were typically admitted for two days. The patients were discharged once the graft was confirmed to be attached, the anterior chamber was stable, and no immediate complications were observed. Follow-up visits were arranged based on the judgment of the surgeon. The follow-up was scheduled at 1 week, 1 month, and subsequently once every three months for the first year. Post-operative treatment included topical antibiotics (third-generation or fourth-generation fluoroquinolones), lubricants, and corticosteroids, with the frequency adjusted by the surgeon, all patients received the same corticosteroid regimen, irrespective of their antiglaucoma medications. Patients with a history of herpetic eye disease received systemic antiviral prophylaxis. Steroid-tapering schedules varied depending on the surgeons. Graft rejection cases were managed with intensive topical steroids along with topical cyclosporine in some cases.

### 2.3. Variables

Data were collected using structured data collection sheets. The variables included demographics, systemic or eye comorbidities (such as retinal diseases, cataract surgery, and previous infection), presence or absence of glaucoma preoperatively, medical or surgical management of glaucoma, lens status and clarity, and pre-operative IOP. The following surgical data were collected: the diagnosis, donor and host graft sizes, and post-operative complications. All complications from the day of surgery until the last follow-up (for successful grafts) or the date of diagnosis of failure (for failed grafts) were documented. Graft rejection was defined as the occurrence of new keratic precipitates, anterior chamber inflammation, or worsening of graft edema in a previously clear graft. This study also recorded whether the graft was detached (partially or completely), whether rebubbling was performed, and any episodes of microbial keratitis (diagnosed clinically and confirmed, when possible, by culture, polymerase chain reaction, or confocal microscopy). Post-operative glaucoma was counted only if it required medical or surgical treatment, and short-term IOP spikes were not included. A persistent epithelial defect was defined as epithelial breakdown lasting >14 days.

### 2.4. Outcome Measures

The primary outcomes of this study were graft survival among patients with glaucoma and the glaucoma-related risk factors for graft failure.

### 2.5. Statistical Analysis

Data were collected, stored, and managed in a spreadsheet using Microsoft Excel 2010. Figure preparation and data analyses were performed using SPSS^®^ version 21.0 (IBM Corp, Armonk, NY, USA). Categorical variables are represented as frequencies and percentages and compared using the chi-square test. Fisher’s test was used when the data comprised fewer than five cells. The normality of continuous variables was determined using the Shapiro–Wilk test and Q-Q plots. The non-normally distributed data are reported as median (interquartile range [IQR]) and compared using the Mann–Whitney test. The risk factors for graft failure were determined using univariate and multivariate Cox regression analyses. Additionally, Kaplan–Meier survival curves were plotted to visualize and estimate the cumulative survival probabilities. Differences were considered significant at *p* < 0.05.

## 3. Results

### 3.1. Demographic and Clinical Characteristics

This study examined the data of 65 eyes from 61 patients with glaucoma who underwent EK. The median age of the patients was 68 years (IQR: 60–76). Most patients were males (*n* = 32; 52.5%) and Saudi nationals (90.2%) (Table 1).

Pre-existing and post-operative glaucoma were noted in 53 (81.5%) and 12 eyes (18.5%), respectively. Medical therapy was required for 48 eyes (73.8%). The most common medical therapies were brimonidine (Alphagan) (45.8%) and dorzolamide and timolol combination (Cosopt) (47.9%). Latanoprost (Xalatan) was used to treat 14 eyes (29.2%). Several patients were on combination therapy, with 22 patients (45.8%) using two medications and 8 patients (16.7%) using ≥3 medications. Surgical intervention was performed on 44 eyes (67.7%), which was mainly trabeculectomy with mitomycin C (45.5%) or Ahmed valve implantation (25%). The proportion of patients who underwent both medical and surgical treatments in the pre-operative glaucoma group was significantly higher than that in the pre-existing glaucoma group (54.7% vs. 8.3%, *p* = 0.004) (Table 2).

### 3.2. Graft Survival Rates

**Figure 1:**
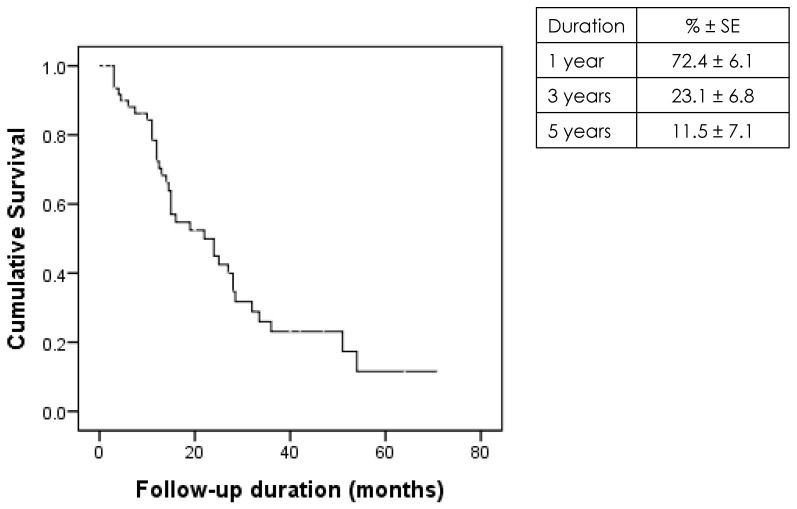
Kaplan–Meier survival curve for graft survival in 65 glaucoma cases.

Kaplan–Meier analysis revealed that the graft survival rates were 72.4% ± 6.1%, 23.1% ± 6.8%, and 11.5% ± 7.1% at 1, 3, and 5 years, respectively (Figure 1).

### 3.3. Antiglaucoma Drop Use and the Risk of Graft Failure

The graft failure rate in the latanoprost group was significantly higher than that in the other treatment group (71.4% vs. 28.6%, *p* = 0.024). Most of these failures were classified as secondary graft failures (80%, *p* = 0.015). Among the graft failure cases, the incidence rate of endothelial rejection episodes was 62.5%. Other antiglaucoma medications, including brimonidine, timolol, and dorzolamide–timolol combination, were not significantly associated with graft failure (Table 3 and Table 4).

Next, the outcomes of eyes treated with latanoprost were compared with those of eyes treated with other regimens. Patients in the latanoprost-treated group were slightly older than those in the other treatment group (median age: 69 vs. 68 years, *p* = 0.633). At the time of surgery, all patients in the latanoprost group were pseudophakic. The incidence rate of secondary graft failure in the latanoprost group was significantly higher than that in the other treatment group (57.1% vs. 26.5%, *p* = 0.043). Other clinical features, including post-operative complications, presence of diabetes, surgical technique, and graft size, were not significantly different between the groups (Table 5).

## 4. Discussion

Recently, EK has become the standard surgical approach for managing endothelial dysfunction. Compared with PK, EK offers several significant advantages, including rapid visual recovery, decreased incidence of post-operative astigmatism, and low overall complication rates [1,19]. Furthermore, previous studies have reported that graft survival rates with EK are up to 79% at 10 years, highlighting the long-term effects of the procedure under optimal conditions [4].

However, glaucoma decreases graft longevity. Patients with glaucoma are reported to be associated with decreased survival rates and increased graft failure rates [3,4,20,21,22,23,24]. This is consistent with the findings of this study, which reported that the graft survival rates were 72.4% ± 6.1, 23.1% ± 6.8, and 11.5% ± 7.1 at 1, 3, and 5 years, respectively, among patients with glaucoma. Several factors contribute to the poor outcomes in patients with glaucoma. Structural and physiological alterations in aqueous humor dynamics, especially glaucoma surgery, may adversely affect graft adhesion, healing, and long-term survival [15,20,25]. These procedures impose additional surgical stress, compromising endothelial health and accelerate endothelial cell loss [8,22,26]. Takemori et al. reported that trabeculectomy was significantly associated with increased endothelial cell loss and graft failure [8]. Moreover, eyes that have undergone trabeculectomy may experience earlier graft failure (25). Kang et al. and Decroos et al. revealed an increased risk of failure in eyes with aqueous shunt devices [10,16]. Nahum et al. reported that the presence of an aqueous shunt increases the risk of repeat surgery [18]. These findings indicate the challenges associated with managing corneal transplantation in eyes with glaucoma.

In addition to the surgical factors, the medical management of glaucoma after endothelial keratoplasty also plays a crucial role. Previous studies have shown that the need for escalation of antiglaucoma therapy following DSAEK ranges from 4% to 47.4% of eyes, reflecting the difficulty in achieving stable intraocular pressure control in these patients [27,28,29]. Such postoperative IOP fluctuations and the requirement for intensified pharmacological treatment may further compromise endothelial cell viability and graft survival.

Latanoprost is a well-established and effective treatment for reducing IOP, offering long-lasting control with minimal systemic effects compared with timolol [30,31,32]. However, its influence on corneal graft health remains uncertain.This study examined the role of the use of topical antiglaucoma medications (latanoprost), an underrecognized but clinically significant variable, in graft rejection after DSAEK. Latanoprost use was significantly associated with graft rejection. Among the eyes treated with latanoprost, the graft success and failure rates were 28.6% and 71.4%, respectively. Additionally, 80% of graft failure cases were classified as secondary graft failures. The incidence of endothelial rejection among graft failure cases was 62.5%. These findings may be explained by the underlying immune mechanisms of post-keratoplasty inflammation. Maharana et al. highlighted that corneal graft rejection results from activation of antigen-presenting cells and T-cell-mediated cytokine cascades once the cornea’s immune privilege is disrupted. Prostaglandin analogues such as latanoprost may further amplify this immune activation by increasing vascular permeability and facilitating leukocyte recruitment, thereby predisposing the graft to inflammatory rejection [33]. In line with this mechanism, previous case reports and observational studies, which reported that prostaglandin analogs, though effective in reducing IOP, compromise corneal graft survival by exacerbating low-grade inflammation. Nouri-Mahdavi et al. described two cases of immunologic graft rejection after the initiation of topical latanoprost [34]. Warwar RE et al. [35] revealed that latanoprost triggers intraocular inflammation as evidenced by the episodes of anterior uveitis and cystoid macular edema. Dvivedi et al. reported a case of severe bilateral herpetic endotheliitis after latanoprost use [36]. Moreover, studies have demonstrated that prostaglandin analogues can disrupt the blood aqueous barrier, leading to increased protein leakage and intraocular inflammation. Miyake et al. reported that latanoprost accelerates disruption of the blood aqueous barrier and increases the incidence of cystoid macular edema in early postoperative pseudophakic eyes [37], while Arcieri et al. confirmed similar findings in pseudophakic and aphakic patients during a 6-month randomized trial [38]. These findings further support the hypothesis that prostaglandin-induced barrier breakdown may facilitate inflammatory mediator entry into the anterior chamber, predisposing the graft to immune rejection.

The proinflammatory effect may be specific to prostaglandin analogs. Other commonly used antiglaucoma agents examined in this study, such as timolol, brimonidine, and dorzolamide–timolol combination, were not significantly associated with graft failure. Thus, glaucoma can compromise long-term graft survival after DSAEK, with latanoprost use being associated with increased risk of graft failure. As this was a retrospective analysis, causality cannot be established. However, the observed patterns of secondary failures and endothelial rejection episodes raise important clinical considerations. The identification of these risks allows ophthalmologists to tailor post-operative glaucoma management strategies, potentially improving the graft outcomes in this vulnerable population.

Additionally, when comparing patients who used latanoprost with those who received other glaucoma treatments, no significant differences in demographic characteristics, including gender distribution, were observed. This indicates that gender was not a contributing factor to the differences in graft survival between the two groups.

This study had some limitations. The results of this study cannot be generalized as this was a retrospective, single-center study with a relatively small cohort, especially in the latanoprost subgroup. Additionally, the details of antiglaucoma medications, such as duration of use and patient compliance, were not consistently available, which may affect the outcomes. Furthermore, the lack of serial endothelial cell count data limited the analysis of endothelial health and the mechanisms of graft failure. Finally, although this study demonstrated that latanoprost use was significantly associated with graft failure, the causality could not be confirmed. Large prospective multicenter studies with standardized reporting are needed to confirm these findings and to better understand the inflammatory pathways linked to prostaglandin use. Future work should also compare different antiglaucoma regimens to identify safer alternatives for post-keratoplasty patients. Establishing clear clinical guidelines for managing glaucoma after endothelial keratoplasty may ultimately help improve long-term graft survival and patient outcomes.

## 5. Conclusions

DSAEK offers a reliable and less invasive option for visual rehabilitation in patients with glaucoma. However, the presence of glaucoma remains a major factor limiting long-term graft survival. In our cohort, graft failure was often secondary and frequently linked to endothelial rejection, particularly among patients using prostaglandin analogues. These findings highlight the need for cautious use of prostaglandins and close postoperative monitoring to maintain graft clarity.

## Figures and Tables

**Table 1:** Demographic characteristics of patients.

Characteristic	ALL (*n* = 61) *n* (%)
Age in years, median (IQR)	68 (60–76)
Gender	
Male	32 (52.5)
Female	29 (47.5)
Nationality	
Saudi	55 (90.2)
Non-Saudi	6 (9.8)

**Table 2:** Glaucoma treatment strategies.

	All	Group I Glaucoma Pre-Operative	Group II Glaucoma Postoperative	*p* Value
Number	65	53	12	----
Medically treated	48 (73.8)	39 (73.6)	9 (75.0)	0.998
Brimonidine (Alphagan)	22 (45.8)	18 (46.2)	4 (44.4)	0.998
Timolol	8 (16.7)	5 (12.8)	3 (33.3)	0.159
Cosopt	23 (47.9)	18 (46.2)	5 (55.6)	0.719
Latanoprost (Xalatan)	14 (29.2)	13 (33.3)	1 (11.1)	0.250
Surgically treated	44 (67.7)	41 (77.4)	3 (25.0)	0.001 *
Trabeculectomy MMC	20 (45.5)	20 (48.8)	0 (0.0)	0.239
Ahmed Valve	11 (25.0)	10 (24.4)	1 (33.3)	0.998
PI	9 (20.5)	7 (17.1)	2 (66.7)	0.101
Both—Medical and surgical	30 (46.2)	29 (54.7)	1 (8.3)	0.004 *

* Statistically significant at 5% level of significance.

**Table 3:** Univariate analysis of glaucoma risk factors associated with graft failure.

Risk Factor	Success (*n* = 37) *n* (%)	Failure (*n* = 28) *n* (%)	*p* Value
Glaucoma			
Pre-operative (*n* = 53)	30 (56.6)	23 (43.4)	0.913
Post-Operative (*n* = 12)	7 (58.3)	5 (41.7)	
Treated medically			
No (*n* = 17)	10 (58.8)	7 (41.2)	0.854
Yes (*n* = 48)	27 (56.3)	21 (43.8)	
Brimonidine (Alphagan) (*n* = 22)	11 (50.0)	11 (50.0)	0.422
Timolol (*n* = 8)	4 (50.0)	4 (50.0)	0.715
Cosopt (*n* = 23)	12 (52.2)	11 (47.8)	0.585
Latanoprost (Xalatan) (*n* = 14)	4 (28.6)	10 (71.4)	0.024 *
Treated surgically			
No (*n* = 21)	12 (57.1)	9 (42.9)	0.980
Yes (*n* = 44)	25 (56.8)	19 (43.2)	

* Statistically significant at 5% level of significance.

**Table 4:** Cases treated with latanoprost (Xalatan).

	Total (*n* = 14) *n* (%)	Success (*n* = 4) *n* (%)	Failure (*n* = 10) *n* (%)	*p* Value
Primary graft failure	2 (14.3)	0 (0.0)	2 (20.0)	0.998
Secondary graft failure	8 (57.1)	0 (0.0)	8 (80.0)	0.015 *
Endothelial rejection	5 (35.7)	0 (0.0)	5 (50.0)	0.221

* Statistically significant at 5% level of significance.

**Table 5:** Subgroup analysis of patients who used latanoprost (Xalatan).

	Latanoprost (Xalatan) (*n* = 14) *n* (%)	Others (*n* = 34) *n* (%)	*p* Value
Age in years, median (IQR)	69 (65–81)	68 (61–78.5)	0.633
Gender			
Female (*n* = 23)	7 (50.0)	16 (47.1)	0.998
Male (*n* = 25)	7 (50.0)	18 (52.9)	
Treated surgically			
No (*n* = 30)	9 (64.3)	21 (61.8)	0.870
Yes (*n* = 18)	5 (35.7)	13 (38.2)	
Developed glaucoma			
No (*n* = 39)	13 (92.9)	26 (76.5)	0.250
Yes (*n* = 9)	1 (7.1)	8 (23.5)	
Lens status			
Pseudophakic (*n* = 47)	14 (100)	33 (97.1)	0.998
Aphakic (*n* = 1)	0 (0.0)	1 (2.9)	
PBK diagnosis			
No (*n* = 16)	4 (28.6)	12 (35.3)	0.746
Yes (*n* = 32)	10 (71.4)	22 (64.7)	
Vascularization			
No (*n* = 46)	13 (92.9)	33 (97.1)	0.503
Yes (*n* = 2)	1 (7.1)	1 (2.9)	
Combined surgery			
No (*n* = 44)	13 (92.9)	31 (91.2)	0.998
Yes (*n* = 4)	1 (7.1)	3 (8.8)	
Post-operative Complications			
No (*n* = 20)	4 (28.6)	16 (47.1)	0.338
Yes (*n* = 28)	10 (71.4)	18 (52.9)	
Lenticule detachment			
No (*n* = 42)	13 (92.9)	29 (85.3)	0.656
Yes (*n* = 6)	1 (7.1)	5 (14.7)	
Rebubbling (*n* = 5)			
No (*n* = 1)	1 (100)	0 (0.0)	0.200
Yes (*n* = 4)	0 (0.0)	4 (100)	
Endothelial rejection			
No (*n* = 39)	9 (64.3)	30 (88.2)	0.099
Yes (*n* = 9)	5 (35.7)	4 (11.8)	
High IOP			
No (*n* = 41)	12 (85.7)	29 (85.3)	0.998
Yes (*n* = 7)	2 (14.3)	5 (14.7)	
Primary failure			
No (*n* = 44)	12 (85.7)	32 (94.1)	0.569
Yes (*n* = 4)	2 (14.3)	2 (5.9)	
Secondary failure			
No (*n* = 31)	6 (42.9)	25 (73.5)	0.043 *
Yes (*n* = 17)	8 (57.1)	9 (26.5)	
Previous grafts			
First (*n* = 36)	11 (78.6)	25 (73.5)	0.998
Repeated (*n* = 12)	3 (21.4)	9 (26.5)	
Surgical technique			
Lens glide technique (*n* = 12)	3 (21.4)	9 (26.5)	0.998
Busin glide technique (*n* = 36)	11 (78.6)	25 (73.5)	
Physicians’ workload			
≥15 grafts (*n* = 45)	13 (92.9)	32 (94.1)	0.998
<15 grafts (*n* = 3)	1 (7.1)	2 (5.9)	
Histopathology for corneal dystrophies			
Fuch’s dystrophy (*n* = 6)	3 (21.4)	3 (8.8)	0.339
No dystrophy and others (*n* = 42)	11 (78.6)	31 (91.2)	
Graft size for donor, Median (IQR)	7.6 (7.5–7.8)	7.8 (7.5–8.0)	0.901
Graft size for host, Median (IQR)	8.0 (7.3–8.0)	7.8 (7.5–8.0)	0.622
Duration of FU, months, Median (IQR)	27.5 (10.8–44.8)	15.0 (9.0–27.3)	0.196
Presence of Diabetes (*n* = 44)			
No (*n* = 22)	5 (45.5)	17 (51.5)	0.728
Yes (*n* = 22)	6 (54.5)	16 (48.5)	

* Statistically significant at 5% level of significance.

## Data Availability

The datasets generated during and/or analyzed during the current study are not publicly available due to legal issues and the policy of our institution.
